# The Role of Secondary Care Networks, Gender, and Race on Primary Caregiver Burden

**DOI:** 10.1093/geroni/igab046.1893

**Published:** 2021-12-17

**Authors:** Jiaming Liang, Maria Aranda

**Affiliations:** University of Southern California, Los Angeles, California, United States

## Abstract

In addition to primary caregivers, many older adults receive care from secondary care networks (SCN), which include family members and friends. Literature rarely considers support that SCN provided to primary caregivers. This study examines: (a) the association between SCN support and primary caregiver burden, and (b) the intersectional effects of gender (male/female)-race (White/Black) identities of primary caregivers on the association. A cross-sectional study using data from 2015 National Health and Aging Trend Study (NHATS) and National Study of Caregiving (NSOC) was conducted. A total of 967 older adults, 967 primary caregivers, and 2253 secondary caregivers were selected. SCN support was measured by (a) care domain overlap, and (b) proportion of caregiving by SCN. Negative binomial regressions on overall and split samples estimated main effects of SCN support and the intersectional effects of gender and race. Both SCN-related variables were associated with primary caregiver burden, but significant three-way interaction was only found between gender, race, and proportion of caregiving by SCN. Black female caregivers reported heaviest burden and having SCN support was associated with lower risk of being burdened. Whereas Black male caregivers reported lightest burden and SCN support was not associated with their perceived burden. Our findings support the positive role of SCN in reducing stress of primary caregivers, and demonstrate that positive impacts of SCN support vary across gender-race groups. The results indicate a strong need for support programs aimed at promoting cooperation among family caregivers for burden reduction, especially families with female and Black primary caregivers.

